# Low plasma fibrinogen levels are associated with poor prognosis in cutaneous angiosarcoma of the head and neck

**DOI:** 10.1111/cas.15037

**Published:** 2021-07-12

**Authors:** Shoichiro Mori, Tomoki Taki, Yoshie Murakami, Toru Urata, Mao Okumura, Honami Akanabe, Aoi Ebata, Satoko Imai, Kenji Yokota, Masashi Akiyama

**Affiliations:** ^1^ Department of Dermatology Nagoya University Graduate School of Medicine Nagoya Japan

**Keywords:** biomarker, guideline, prognostic factor, soft tissue sarcoma, TNM staging

## Abstract

Angiosarcoma of the head and neck (ASHN) is one of the most aggressive malignancies of the skin, but the prognostic factors are not well known because of its rarity. Recently, high plasma fibrinogen levels were reported to predict poor prognosis in several malignancies. In the present retrospective study, we suggest that low plasma fibrinogen levels predict poor prognosis for ASHN.

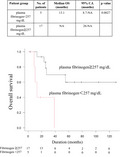

## 1 INTRODUCTION

Angiosarcomas are malignant vascular tumors that account for 2% of all soft tissue sarcomas.^1^ Fifty percent cases of cutaneous angiosarcoma occur in the head and neck.^1^ The 5‐year survival rate for cutaneous angiosarcoma is 10%‐20%.^2^, ^3^ Due to the rarity of angiosarcomas, the prognostic factors are seldom reported. Plasma fibrinogen levels have been reported as a prognostic factor for several malignancies, including soft tissue sarcomas.^4^, ^5^ Here, we investigated plasma fibrinogen levels as a prognostic factor for angiosarcoma of the head and neck (ASHN).

## 2 MATERIALS AND METHODS

This retrospective study reviewed a cohort of patients histologically diagnosed with ASHN at Nagoya University Hospital between April 2006 and July 2019. A total of 22 patients were enrolled. It was approved by the institutional review board of the Nagoya University Graduate School of Medicine. Data were obtained from clinical medical records, which included the following: age, sex, ECOG performance status (PS) score, primary site, tumor size (T stage), lymph node status (N stage), and distant metastasis (M stage), follow‐up time outcome, treatment, and pretreatment laboratory data. The TNM staging followed the Japanese Dermatological Association (JDA) guidelines for ASHN (Tables 1 and S1). The optimal cut‐off value of plasma fibrinogen levels was determined by the receiver operating curves for overall survival (OS). After determining the optimal cut‐off value, we classified the patients into two groups by plasma fibrinogen levels. The patients were categorized into a high plasma fibrinogen group (plasma fibrinogen levels equal to or above the cut‐off value) and a low plasma fibrinogen group (plasma fibrinogen levels below the cut‐off value). The distributions of the continuous variables were summarized as median and interquartile range. The distributions of the categorical variables are described as frequencies and percentages. The Mann‐Whitney *U* test and Fisher’s exact test were used to compare continuous variables and categorical variables, respectively. Overall survival was assessed using the Kaplan‐Meier method. Differences between groups were estimated using the log‐rank test. Univariable Cox proportional hazard models were used to evaluate the association between plasma fibrinogen levels and OS. All statistical analyses were undertaken with EZR.^6^ All analyses used the conventional *P* value of <.05 for significance.

**TABLE 1:** Tumor‐node‐metastasis staging for scalp angiosarcoma from the Japanese Dermatological Association Guidelines for Scalp Angiosarcoma

T	Primary tumor
TX	Cannot be assessed
T1	Smaller than or equal to 5 cm in maximum diameter
T2	Larger than 5 cm in maximum diameter, or multiple lesions, or facial invasion
T3	With skull base invasion

N	Regional lymph nodes
NX	Cannot be assessed
N0	No regional lymph node metastasis
N1	Regional lymph node metastasis

M	Distant metastasis
MX	Cannot be assessed
M0	No distant metastasis
M1	Distant metastasis

TNM staging			
**I**	T1	N0	M0
**Ⅱ**	T2	N0	M0
**Ⅲa**	T3	N0	M0
**Ⅲb**	Any T	N1	M0
**Ⅳ**	Any T	Any N	M1

## 3 RESULTS

The baseline characteristics of the 22 patients with ASHN included in the present study are shown in Table 2. The cut‐off value of plasma fibrinogen levels was determined to be 257 mg/dL. The high plasma fibrinogen group consisted of 17 patients whose plasma fibrinogen levels equaled or exceeded 257 mg/dL; the low plasma fibrinogen group had five patients whose plasma fibrinogen levels were below 257 mg/dL. There were no statistically significant differences in age, sex, ECOG‐PS score, primary site, outcome, follow‐up duration, or treatment (Table 2).

**TABLE 2:** Clinical features of 22 patients with angiosarcoma of the head and neck: comparison between patients with high plasma fibrinogen (≥257 mg/dL) and low plasma fibrinogen (<257 mg/dL)

Clinical feature	Patient group			*P* value*
	Total	High plasma fibrinogen group (≥257 mg/dL)	Low plasma fibrinogen group (<257 mg/dL)	
Total no. of patients (%)	22 (100)	17 (77)	5 (23)	
Age (y)				
Median (range)	76 (62‐86)	76 (62‐86)	76 (68‐81)	.813
IQR	68.5, 79	67, 79	72, 79	
Sex	No. of patients (%)			
Male	15 (68)	10 (59)	5 (100)	.135
Female	7 (32)	7 (41)	0 (0)	
ECOG‐PS score				
0‐1	19 (86)	15 (88)	4 (80)	1.000
2‐4	3 (14)	2 (12)	1 (20)	
Primary site				
Head	21 (95)	16 (94)	5 (100)	1.000
Face/neck	1 (5)	1 (6)	0 (0)	
T stage				
T1	6 (27)	4 (24)	2 (40)	.585
T2	16 (73)	13 (76)	3 (60)	
T3	0 (0)	0 (0)	0 (0)	
N stage				
N0	20 (91)	16 (94)	4 (80)	.411
N1	2 (9)	1 (6)	1 (20)	
M stage				
M0	21 (95)	16 (94)	5 (100)	1.000
M1	1 (5)	1 (6)	0 (0)	
TNM stage				
Ⅰ	3 (14)	2 (12)	1 (20)	.489
Ⅱ	16 (73)	13 (76)	3 (60)	
Ⅲa	0 (0)	0 (0)	0 (0)	
Ⅲb	2 (9)	1 (6)	1 (20)	
Ⅳ	1 (5)	1 (6)	0 (0)	
Outcome				
Alive	13 (59)	12 (71)	1 (20)	.116
Dead	9 (41)	5 (29)	4 (80)	
Months of follow‐up				
Median (range)	26.1 (2.3‐129.7)	27.0 (2.3‐129.7)	17.8 (8.7‐38.7)	.0583
IQR	14.4, 55.8	23.4, 58.0	10.3, 18.2	
Treatment	No. of patients (%)			
Surgery	11 (50)	9 (53)	2 (40)	.939
Radiation	20 (91)	15 (88)	5 (100)	
IL‐2	7 (32)	5 (29)	2 (40)	
Paclitaxel	17 (77)	13 (76)	4 (80)	
Docetaxel	5 (23)	4 (24)	1 (20)	
Pazopanib	6 (27)	6 (35)	0 (0)	
Eribulin	2 (9)	2 (12)	0 (0)	
Doxorubicin	1 (5)	1 (6)	0 (0)	

Abbreviations: IL‐2, interleukin‐2; IQR, interquartile range; PS, performance status.

* Statistically significant: *P* < .05.

In the present study cohort, male patients predominated (68%). All the patients in the low plasma fibrinogen group were male (5/5). The mortality rate was 41%, 29%, and 80% for the total, the high plasma fibrinogen group, and the low plasma fibrinogen group, respectively. The median values (ranges) for follow‐up period were 26.1 (2.27‐129.7) months, 27.0 months (2.3‐129.7), and 17.8 months (8.7‐38.7) for the total, high plasma fibrinogen group, and low plasma fibrinogen group, respectively.

**FIGURE 1:**
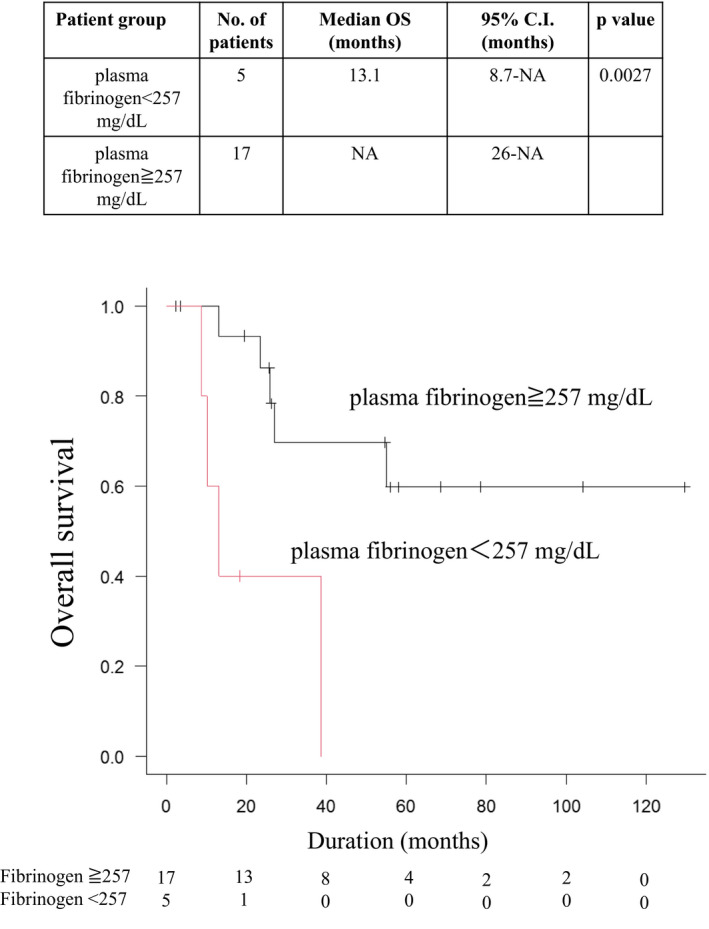
Kaplan‐Meier method to determine overall survival (OS) rates in 22 patients with angiosarcoma of the head and neck. The OS rate is significantly higher in the high plasma fibrinogen group than in the low plasma fibrinogen group (*P* = .0027)

The Kaplan‐Meier curve showed the OS rate to be significantly higher in the high plasma fibrinogen group than in the low plasma fibrinogen group (Figure 1) (*P* = .0027). The univariable model showed that patients with low plasma fibrinogen levels had a significantly poor OS (hazard ratio 6.78; 95% confidence interval, 1.63‐28.2; *P* = .008503].

## 4 DISCUSSION

The prognostic factors for ASHN are not well known because of its rarity. Recently, high plasma fibrinogen levels were reported to predict poor prognosis in several malignancies.^7^ For example, a meta‐analysis revealed high pretreatment plasma fibrinogen levels to be associated with poor OS in colorectal cancer.^7^ The production of plasma fibrinogen is induced by inflammation.^8^ It is possible that inflammation from advanced malignancies secondarily causes hyperfibrinogenemia. Unexpectedly, the present study suggests that low plasma fibrinogen levels predict poor prognosis. This result is the exact opposite of the trends previously reported for other malignancies. Angiosarcoma of the head and neck is an extremely hemorrhagic tumor. From repeated microbleeds and blood coagulation in ASHN lesions, plasma fibrinogen is considered to be consumed and to decrease in patients with ASHN. Therefore, we speculate that the reduced plasma fibrinogen levels might reflect tumor burden indirectly.

The JDA guidelines for ASHN have been used for TNM staging. The T stage depends on the maximum diameter of the lesions, the number of lesions (single or multiple), and the presence/absence of face invasion and bone invasion. However, the shapes of the lesions are often irregular, and the maximum diameter of the lesions does not always reflect the exact size of the lesions. Additionally, the lesions are sometimes mixed with senile purpura, so it can be difficult to define the T stage. Angiosarcoma of the head and neck often metastasizes to the lung, causing fatal hemopneumothorax. To make matters worse, lesions from the lung metastasis of ASHN sometimes do not present typical nodules in computed tomography (CT) scans.^9^, ^10^ Tateishi et al^9^ investigated 24 metastatic angiosarcomas of the lung and reported that multiple solid nodular lesions, multiple thin‐walled cysts, and ground‐glass attenuation surrounding thin‐walled cystic lesions were seen in 63%, 21%, and 17% of cases, respectively. Cystic lesions of the lung are also seen in emphysema, and ground‐glass attenuation is also seen in pneumonia. Thus, it is often difficult to define whether these findings in CT scans are lung metastases of ASHN.^10^ Considering the difficulty of appropriate staging, it is very important to identify the factors that reflect the disease status. Plasma fibrinogen levels might be a promising biomarker for disease progression.

As a limitation, the present study is a single‐center, retrospective study with a small number of cases.

In conclusion, the present study suggests that pretreatment plasma fibrinogen levels are a useful prognostic factor for ASHN that could reflect tumor burden. Further large‐scale investigations are needed to establish the plasma fibrinogen levels in patients with ASHN as a prognostic biomarker.

## DISCLOSURE

Masashi Akiyama was provided a scholarship from Maruho Co. Ltd. The other authors have no conflict of interest.

## IRB APPROVAL STATUS

Reviewed and approved by the ethics committee of Nagoya University Hospital (2020‐0128).

## Supporting information

Table S1
