# Supplementary material for: Low plasma fibrinogen levels are associated with poor prognosis in cutaneous angiosarcoma of the head and neck
Source: Cancer Sci. 2021 Jul 12;112(9):3924–7. doi: 10.1111/cas.15037 (PMC8409292; doi:10.1111/cas.15037)
Supplement: Supplementary file 1 — Table S1 [file CAS-112-3924-s001.docx]

**Table S1. Clinical information of 22 patients with ASHN**

| Patient No. | Sex | Metastasis at initial diagnosis | Plasma fibrinogen (mg/dL) | Prognosis | Cancer-specific death? | Recurrence  /metastasis | Treatment | OS  (months) | PFS  (months) |
| --- | --- | --- | --- | --- | --- | --- | --- | --- | --- |
| 1 | M | No | <257 | Alive | no | none | RT (60Gy), PTX | 17 | 17 |
| 2 | M | No | <257 | dead | yes | local | IL-2, surgery, DTX, palliative RT (20Gy) | 12 | 10 |
| 3 | M | No | <257 | dead | yes | LN | surgery, IL-2, RT (53.5Gy), DTX | 38 | 25 |
| 4 | M | No | <257 | dead | yes | bone | RT (60Gy), DTX | 10 | 4 |
| 5 | M | No | <257 | dead | yes | local | RT (60Gy), PTX | 8 | 4 |
| 6 | F | No | ≧257 | Alive | no | none | RT (60Gy), surgery, PTX | 25 | 25 |
| 7 | M | No | ≧257 | Alive | no | none | RT (60Gy), PTX | 55 | 55 |
| 8 | M | No | ≧257 | dead | yes | LN | RT (70Gy), IL-2, surgery, PTX | 23 | 5 |
| 9 | F | No | ≧257 | Alive | no | none | RT (60Gy), PTX | 57 | 57 |
| 10 | M | No | ≧257 | dead | yes | lung | RT (60Gy), PTX, Pazo, DOX | 26 | 10 |
| 11 | F | No | ≧257 | Alive | no | none | RT (60Gy), PTX | 3 | 3 |
| 12 | M | No | ≧257 | Alive | no | lung | RT (60Gy), surgery, PTX, DTX, Pazo | 25 | 11 |
| 13 | F | No | ≧257 | dead | yes | LN | RT (60Gy), surgery, IL-2, RT, PTX, Pazo, | 54 | 23 |
| 14 | M | lung, LN | ≧257 | Alive | no | lung, LN | RT (60Gy), PTX | 19 | 3 |
| 15 | M | No | ≧257 | Alive | no | local | RT (60Gy), PTX, Pazo, Eri | 53 | 26 |
| 16 | F | No | ≧257 | Alive | no | LN | surgery, RT (60Gy), DTX, Pazo | 67 | 24 |
| 17 | M | No | ≧257 | Alive | yes | local | surgery, RT (66Gy), DTX, IL-2, PTX | 127 | 4 |
| 18 | F | No | ≧257 | dead | unknown | none | surgery, DTX | 12 | 12 |
| 19 | M | No | ≧257 | dead | yes | local | RT (60Gy), PTX, DTX | 25 | 10 |
| 20 | M | No | ≧257 | Alive | no | non | PTX | 2 | 2 |
| 21 | M | No | ≧257 | Alive | no | local | surgery, RT (60Gy), IL-2, PTX, Pazo, Eri | 102 | 55 |
| 22 | F | No | ≧257 | Alive | no | local | RT (60Gy), surgery, IL2 | 77 | 32 |

OS, overall survival; M, male; F, female; PFS, progression-free survival; RT, radiation therapy; PTX, paclitaxel; IL-2, interleukin-2; DTX, docetaxel; LN, lymph node; Pazo, pazopanib; DOX, doxorubicin; Eri, eribulin
